# Mitochondrial depolarization stabilizes the vitamin B_12_ chaperone MMADHC in the cytosol to increase MTR activity

**DOI:** 10.1073/pnas.2537797123

**Published:** 2026-09-08

**Authors:** Sneha P. Rath, Zhu Li, Arkajit Guha, Fangcong Dong, Ruma Banerjee, Vamsi K. Mootha

**Affiliations:** ^a^ Department of Molecular Biology, Mass General Hospital, Boston, MA 02114; ^b^Department of Systems Biology and Medicine, Harvard Medical School, Boston, MA 02115; ^c^ Broad Institute of Massachusetts Institute of Technology and Harvard, Cambridge, MA 02142; ^d^ Department of Biological Chemistry, University of Michigan, Ann Arbor, MI 48109; ^e^HHMI, Boston, MA 02114

**Keywords:** vitamin B_12_, proton motive force, mitochondria, methionine synthase, MMADHC

## Abstract

Of the ~1,100 mitochondrial proteins, only a handful like PINK1 and ATFS-1 are known to stabilize and relocalize upon collapse of the proton motive force (PMF) to execute signaling roles. To systematically identify genes that increase exclusively at the protein level upon PMF collapse, we performed a joint proteomic and RNA-seq screen. The screen revealed 10 candidates (six mitochondrial), including two genes in vitamin B_12_ metabolism - the B_12_ chaperone MMADHC and cytosolic B_12_-dependent 5-methyltetrahydrofolate-homocysteine methyltransferase (MTR). MMADHC is short-lived across cell types and we show that its levels increase with PMF collapse. MMADHC stabilization precedes PINK1 activation in a time course of increasing mtDNA depletion, suggesting greater sensitivity to PMF collapse. MMADHC accumulates in mitochondria with LONP1 inhibition but in the cytosol upon PMF collapse, likely due to mitochondrial import failure. Cytosol-stabilized MMADHC increases MTR levels and activity. Altogether, the mitochondrial PMF regulates the cytosolic B_12_-dependent MTR, integral to one-carbon metabolism, by controlling the stability and compartmentalization of the B_12_ chaperone MMADHC.

## Significance

Humans have only two vitamin B_12_-dependent enzymes—mitochondrial methylmalonyl-CoA mutase and cytosolic 5-methyltetrahydrofolate-homocysteine methyltransferase (MTR)—and both require a common B_12_ chaperone MMADHC. We show that MMADHC is a low abundant, short-lived protein that is continuously imported and degraded by energized mitochondria. Upon collapse of the mitochondrial proton motive force (PMF), MMADHC accumulates in the cytosol and increases the levels and activity of MTR, critical for one-carbon metabolism. This PMF-dependent regulation of MMADHC stability and localization is important for understanding cofactor rationing and spatiotemporal compartmentalization of B_12_ metabolism.

The mitochondrial proteome comprises ~1,100 nuclear-encoded proteins (1) that are translated on cytosolic ribosomes and imported into mitochondria. Depending on the final submitochondrial destination, protein import requires chaperones, redox recycling, proton motive force (PMF), and ATP (2). Thus, the mitochondrial proteome, which represents ~5% of the total human proteome, is poised to sense mitochondrial proteostasis, redox, and bioenergetic status simply by virtue of its dependence on import.

Three prominent examples where cells exploit the opportunity of import-dependent mitochondrial surveillance and couple it to signaling are PINK1, *Caenorhabditis elegans* ATFS-1, and DELE1. PINK1 and ATFS-1 constantly undergo PMF-dependent degradation: PINK1 is clipped by PARL (3) during its transit into the mitochondrion and released to the cytosol for proteasomal degradation, whereas ATFS-1 is degraded by LONP1 (4) in the mitochondrial matrix. At a critical threshold of PMF collapse, both proteins fail to import, and while PINK1 accumulates on the outer mitochondrial membrane (OMM) to initiate mitophagy (5), ATFS-1 translocates to the nucleus to activate the mitochondrial unfolded protein response (6). More recently, DELE1 was also shown to sense PMF collapse, as well as other stresses, during transit through the outer and inner mitochondrial membranes, which causes its mitochondrial release and subsequent activation of the cytosolic integrated stress response kinase HRI (7–9). An open question is whether other mitochondrial proteins exhibit similar PMF-dependent regulation and the impact of their conditional extramitochondrial sequestration or retrotranslocation.

Here, we performed a multiomic screen to systematically identify mitochondrial proteins that are stabilized upon PMF collapse. Our analysis spotlights MMADHC, a vitamin B_12_ chaperone. Humans have two B_12_-dependent enzymes: i) cytosolic 5-methyltetrahydrofolate-homocysteine methyltransferase (MTR) and ii) mitochondrial methylmalonyl-CoA mutase (MMUT) (10). MTR converts methyl-tetrahydrofolate, the circulating form of folate, to tetrahydrofolate (THF) which is required for nucleotide biosynthesis, and methylates homocysteine to produce methionine in the process (11). MMUT converts methylmalonyl-CoA to succinyl-CoA, catalyzing the final step in the TCA anaplerosis of valine, methionine, isoleucine, threonine and odd-chain fatty acids (12). MMADHC is genetically linked to both MTR and MMUT based on patient mutations that lead to a toxic accumulation of their respective substrates, causing either isolated or combined homocystinuria and methylmalonic aciduria (13). Human MMADHC has an N-terminal mitochondrial targeting sequence (MTS) and is a 296 amino acid long protein, the first third of which is predicted to be disordered while the last two-thirds belongs to the ferredoxin nitroreductase superfamily and coordinates B_12_ (14). While the role of MMADHC in the mitochondrial B_12_ pathway remains elusive, previous studies have demonstrated that it can load the B_12_ cofactor onto the cytoplasmic target MTR (15). Whether and how the mitochondrial vs. cytoplasmic compartmentalization of MMADHC is regulated remains largely unexplored.

Using an unbiased approach, our study uncovered that MMADHC is regulated by the mitochondrial PMF. We show that MMADHC is continuously degraded by LONP1 in energized mitochondria but upon PMF collapse it is retained in the cytosol where it increases MTR levels and activity. Thus, the PMF regulates MMADHC stability and compartmentalization and, in turn, cytosolic MTR activity, with implications for one-carbon metabolism.

## Results

### A Multiomic Screen Identifies MMADHC as the Top Hit that Increases in Response to Mitochondrial PMF Collapse.

**Fig. 1.**
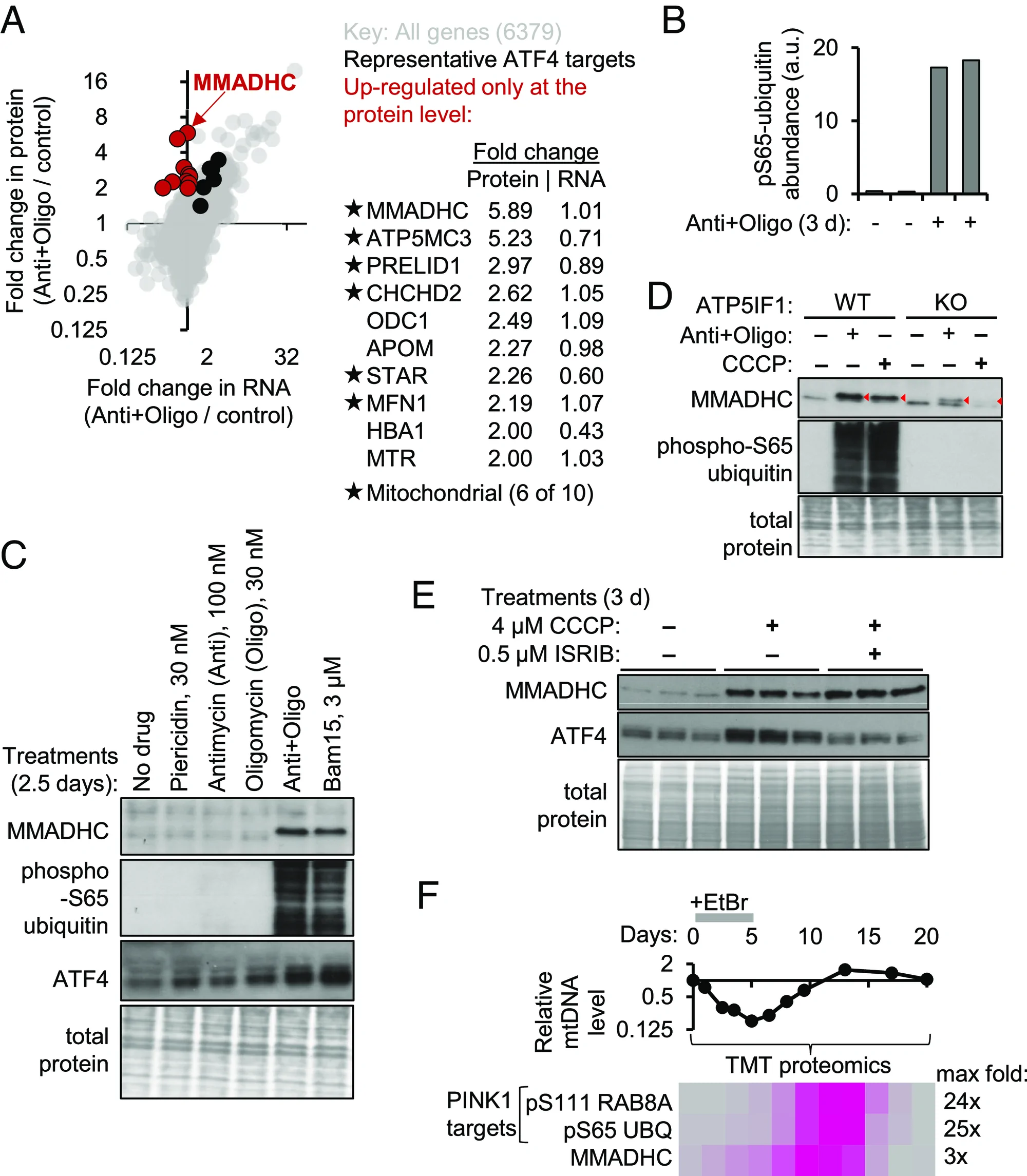
A multiomic screen identifies that MMADHC increases with collapse of the mitochondrial PMF. (*A*) Scatter plot of average fold change in protein vs. RNA levels measured by TMT proteomics and RNAseq respectively in K562 cells treated with antimycin and oligomycin (anti+oligo) for 3 d, in two biological replicates. Black points show representative ATF4 target genes ASNS, CTH, PHGDH, PSAT1, PSPH, TRIB3. Red points are genes with fold change in protein >2 and RNA <1.2-fold and are listed in the table adjacent to the scatter plot. (*B*) Abundance of the PINK1-dependent phosphosite pSer65-ubiquitin detected by TMT proteomics in the samples in *A*. (*C*) Western blot analysis of the indicated target genes in K562 cells treated with electron transport chain (ETC) inhibitors piericidin, antimycin, or oligomycin, targeting complexes I, III, and V respectively, or drugs that collapse the PMF, anti+oligo or the protonophore BAM15. Blot is representative of three biological replicates. (*D*) Western blot analysis of MMADHC and phospho-S65 ubiquitin in sgGFP or sgATP5IF1 K562 cells treated with antimycin and oligomycin (anti+oligo) or uncoupler carbonyl cyanide 3-chlorophenylhydrazone (CCCP) for 3 d. Total protein stained with Coomassie for loading control. Red arrowhead points to the correct band corresponding to MMADHC. Blot is representative of two biological replicates. (*E*) Western blot analysis of MMADHC and ATF4 in K562 cells treated with CCCP or cotreated with CCCP and ISRIB in three biological replicates. Total protein stained with Coomassie for loading control. (*F*) QPCR for mtDNA normalized to nuclear DNA in K562 cells treated with ethidium bromide (EtBr) for 5 d, followed by EtBr-withdrawal for 15 d. Row-scaled heatmap of protein levels of MMADHC vs. two PINK1 target phosphosites quantified by TMT proteomics at the same timepoints as mtDNA analysis.

To systematically identify genes that are upregulated only at the protein level in response to a collapse of the mitochondrial PMF, we performed TMT proteomics and RNAseq analysis of K562 cells treated with antimycin and oligomycin (“anti+oligo”) for 3 d. Of the 6,378 genes with combined protein and RNA level data, only 10 genes were up-regulated exclusively at the protein level (cutoff >twofold). Six of these are mitochondrial proteins, including CHCHD2, which was previously reported to increase at the protein level upon mitochondrial depolarization (16) (Fig. 1*A* and Dataset S1). In contrast, ATF4 target genes were induced both at the RNA and protein level, as expected for a transcriptional response. Although PINK1 was not detected, we observed a robust increase in the PINK1-dependent phosphosite pSer65-ubiquitin (17) in response to anti+oligo (Fig. 1*B*). We focused on the top hit that increased only at the protein level, the vitamin B_12_ chaperone MMADHC, and note that the cytosolic B_12_-dependent MTR, which interacts physically with MMADHC (13), is also a hit in our screen.

To distinguish whether MMADHC is upregulated in response to ETC inhibition or mitochondrial PMF collapse, we compared MMADHC levels across cells treated with a variety of mitochondrial poisons. MMADHC levels did not increase in response to inhibitors of ETC complexes I, III, or V (piericidin, antimycin A, and oligomycin respectively, Fig. 1*C*). MMADHC was up-regulated in response to anti+oligo as well as the mitochondria-specific protonophore BAM15 (Fig. 1*C*). Both these treatments collapsed the PMF as confirmed by accumulation of a PINK1 target, phospho-S65 ubiquitin (Fig. 1*C*). In contrast to MMADHC, ATF4 was induced to varying degrees in all treatments, as expected (18, 19) (Fig. 1*C*).

To further confirm that the increase in MMADHC is responsive to PMF collapse, we tested the effect of ATP5IF1 knockout (KO). Loss of ATP5IF1 defends the PMF by allowing unhindered complex V ATPase activity when the membrane depolarization exceeds a certain threshold (20). Control cells treated with anti+oligo or uncoupler carbonyl cyanide 3-chlorophenylhydrazone (CCCP) for 3 d showed the expected increase in MMADHC and the PINK1 target phospho-S65 ubiquitin, both of which were strongly blunted or prevented altogether in ATP5IF1 KO cells (Fig. 1*D*).

The increase in MMADHC exclusively at the protein level might be due to enhanced translation or decreased proteolysis—mechanisms which are known to regulate ATF4 and PINK1 respectively. In ATF4, ribosomes engage at the conserved upstream open reading frames (uORFs), while the downstream ORF is translated only upon eIF2α phosphorylation, which limits translation initiation and promotes ribosome scanning past the uORFs (21). In principle, human MMADHC could be a candidate for such regulation because it has an uORF that is conserved in *Mus musculus*, *Xenopus laevis*, *Drosophila melanogaster*, as well as the sea slug *Aplysia californica* (*SI Appendix*, Fig. S1). However, ISRIB treatment to rescue translation despite eIF2α phosphorylation (22) blunted induction of ATF4, but not that of MMADHC, in response to the mitochondrial uncoupler CCCP (Fig. 1*E*), ruling out an ATF4-like translational upregulation as a mechanism for MMADHC regulation.

Next, we compared the kinetics and PMF threshold at which MMADHC vs. PINK1/PINK1-dependent phosphosites appear. Proteomics analysis of K562 cells during a time course of ethidium bromide (EtBr) treatment (23), which depletes mtDNA, followed by EtBr withdrawal to recover mtDNA copy number, revealed that both the onset and peak of increase in MMADHC preceded that of the PINK1 targets, phosphosites pS111 on RAB8A and pS65 on UBQ (Fig. 1*F*). In a time course of anti+oligo treatment, MMADHC accumulates robustly within 4 h while the PINK1-dependent pS65 UBQ increases gradually from 4 to 25 h (*SI Appendix*, Fig. S2*A*). In a dose–response of CCCP for a fixed time, MMADHC, PINK1, and pS65 UBQ are detected at comparable doses of CCCP (*SI Appendix*, Fig. S2*B*). These data together reveal that MMADHC, like PINK1, is highly sensitive to PFM collapse, but with faster kinetics.

### MMADHC Is Among the Shortest-Lived Proteins in Multiple Cell Types.

**Fig. 2.**
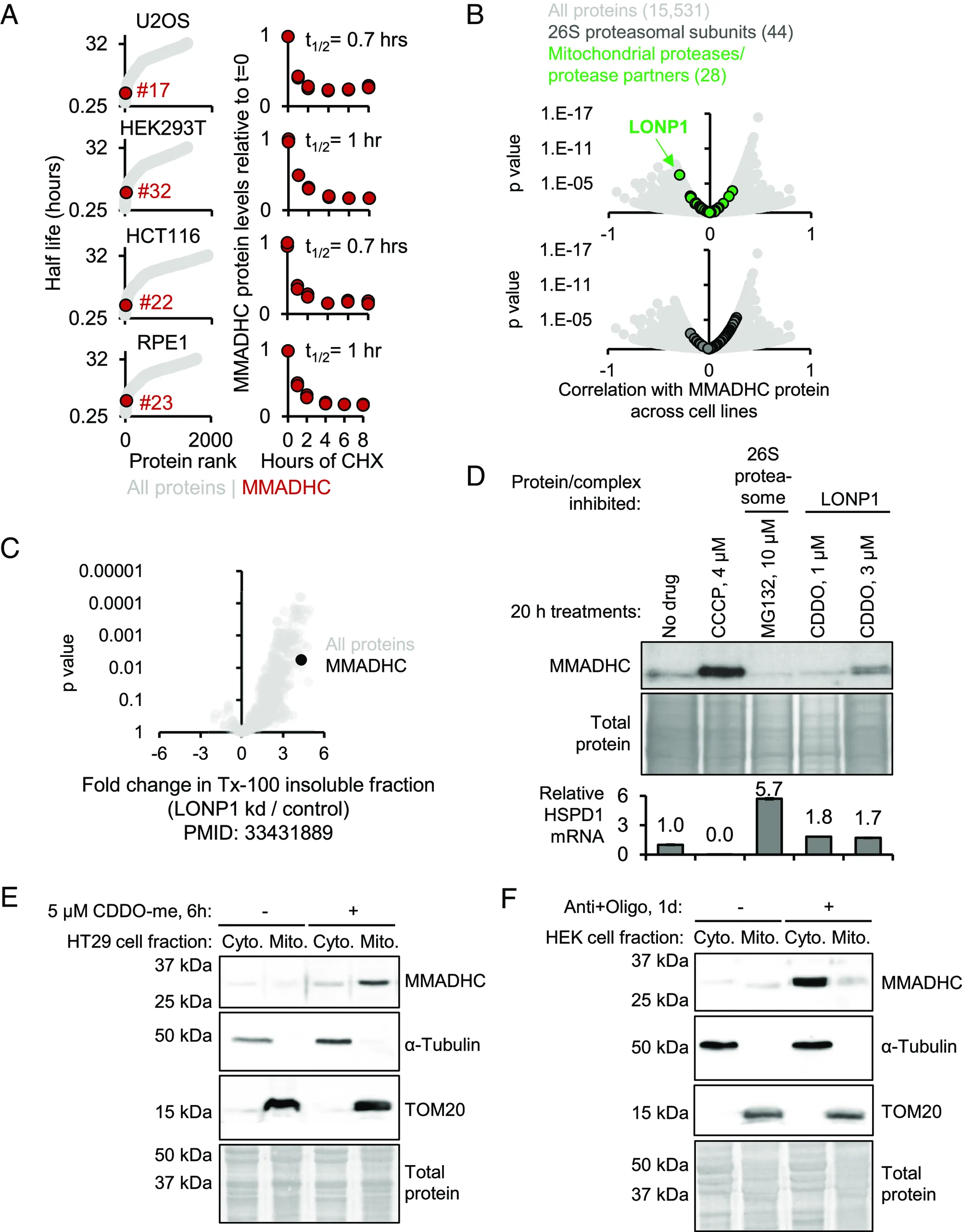
MMADHC is a short-lived protein that accumulates in mitochondria with LONP1 inhibition and in the cytosol with mitochondrial depolarization. (*A*) Protein half-lives (*Left*) and MMADHC levels (*Right*) measured by cycloheximide (CHX) chase in four cell lines. Reanalysis of data from PMID: 34626566. (*B*) Volcano plots of MMADHC protein correlated to all other proteins across Cancer Cell Line Encyclopedia (CCLE). Reanalysis of TMT proteomics data from PMID: 31978347. (*C*) Volcano plot of fold change in protein levels in the triton-100 insoluble fraction in response to LONP1 knockdown. Reanalysis of raw data from PMID 33431889. (*D*) Western blot of MMADHC in K562 cells treated with CCCP, proteasomal inhibitor MG132, and LONP1 inhibitor CDDO-me. Total protein is stained with Coomassie for loading control. QPCR quantification of HSPD1 mRNA relative to ACTB as a proxy of proteotoxic stress. (*E*) Western blot of cytosolic and mitochondrial fractions of HT29 cells treated with LONP1 inhibitor CDDO-me. Total protein is stained with Ponceau for loading control. Blot is representative of three biological replicates. (*F*) Western blot analysis of cytosolic and mitochondrial fractions of HEK293 cells treated with antimycin and oligomycin for 1 d. Blot is representative of three biological replicates.

Analysis of previously published proteome-wide half-lives (24) from cycloheximide pulse and proteomics experiments indicates that MMADHC, with a half-life of ≤1 h, is among the top 50 shortest-lived proteins across all four cell lines/lineages tested (Fig. 2*A*). Notably, three other hits from our screen (Fig. 1*A*) are also short-lived in at least one cell line: PRELID1 (<1 h), ODC1 (<1 h), and CHCHD2 (~2 h). Our joint RNA-seq and TMT proteomics of K562 cells revealed that, although MMADHC mRNA is highly expressed, the steady-state levels of the protein are low. Thus, while *MMADHC* mRNA ranks 258th, the protein ranks 4445th out of 6,378 genes ranked in descending order of RNA or protein expression respectively (*SI Appendix*, Fig. S3 *A* and *B*). There is a strong correlation between mRNA and protein levels across the 6,378 genes with joint data (Spearman correlation: 0.38, *P* value: 1.1E-222). For example, subunits of the cytosolic ribosome are highly expressed at both the RNA and the protein level in contrast to MMADHC (*SI Appendix*, Fig. S3*C*).

### MMADHC Accumulates in Mitochondria Upon LONP1 Inhibition but in the Cytosol Upon Mitochondrial Depolarization.

We hypothesized that MMADHC might be regulated like PINK1 or ATFS-1, which are constantly imported and degraded by PARL/proteasome or LONP1 respectively and stabilized only when they fail to import due to mitochondrial depolarization. To test this hypothesis, we first sought to identify the protease responsible for MMADHC turnover.

We performed correlation analysis of MMADHC against each of ~15,000 detected proteins across the CCLE (25). Out of the 28 detected mitochondrial proteases, LONP1 is the sole protease that is significantly negatively correlated with MMADHC while in contrast, the cytosolic proteasomal subunits do not show any significant correlation with MMADHC (Fig. 2*B*). Breaking down the CCLE analysis by lineage, MMADHC is negatively correlated with LONP1 within several lineages (*SI Appendix*, Fig. S4*A*), most notably in the ovarian and colon lineages (*SI Appendix*, Fig. S4*B*).

Reanalysis of published proteomics (26) of the Triton-100 insoluble fraction from control vs. LONP1 knockdown cells revealed that MMADHC was significantly enriched in the insoluble fraction upon LONP1 knockdown (Fig. 2*C*), consistent with a putative role of LONP1 in degrading MMADHC and preventing its aggregation in mitochondria. Chemical inhibition of LONP1 by CDDO-me increased MMADHC in K562 cells, but proteasomal inhibition by MG132 did not (Fig. 2*D*). Our data are consistent with the prior observation that LONP1 inhibition increased MMADHC in H1944 cells (27).

Next, we determined MMADHC localization in response to LONP1 inhibition vs. mitochondrial depolarization. Because CDDO-me treatment decreased K562 cell viability, we tested its effect in a different cell line, HT-29, selecting from the colon lineage where LONP1 is strongly and negatively correlated with MMADHC (*SI Appendix*, Fig. S3*B*). In HT-29 cells treated with CDDO-me, MMADHC increased and was enriched in the mitochondrial fraction (Fig. 2*E*), consistent with MMADHC being degraded primarily by LONP1 in the mitochondrial matrix. In contrast, MMADHC accumulated in the cytosol upon mitochondrial depolarization by anti+oligo (Fig. 2*F*). Thus, MMADHC is regulated in a PINK1-like manner in that its turnover depends on a mitochondrial protease and PMF and it is stabilized in the cytosol following PMF collapse.

### Cytoplasmic Stabilization of MMADHC Increases MTR Protein and Activity.

**Fig. 3.**
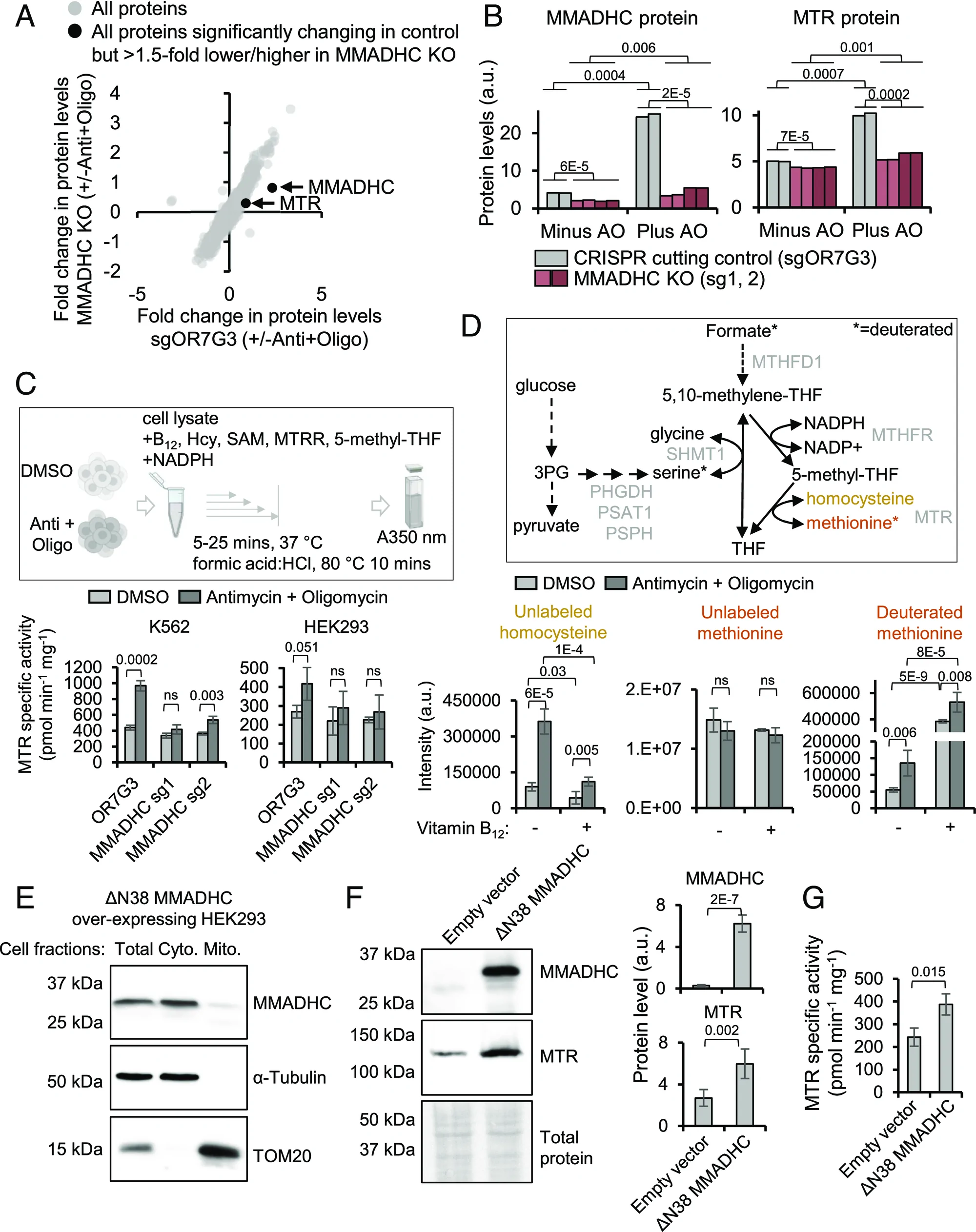
Cytosol-stabilized MMADHC increases MTR protein and activity. (*A*) Scatter plot of log_2_ fold change in protein levels of MMADHC KO (sg1, sg2 combined) vs. control (sgOR7G3 CRISPR cutting control) cells treated with antimycin and oligomycin (anti+oligo) for 3 d and analyzed by TMT proteomics. Average log_2_ fold change from two replicates for control (sgOR7G3) and four replicates across two separate sgRNAs for MMADHC are plotted. (*B*) Bar plots of MMADHC and MTR levels from the experiment shown in (*A*). (*C*) Schematic of MTR activity assay in cell extracts. MTR specific activity measured in extracts of K562 and HEK293 cells treated with or without antimycin and oligomycin for 3 d. Error bars represent SD across three biological replicates. (*D*) Schematic of deuterated formate tracing. Quantification of MTR substrate homocysteine and product methionine (unlabeled and deuterated) by mass spectrometry in K562 cells treated with or without antimycin and oligomycin for 3 d and labeled with fully deuterated formate in the presence or absence of vitamin B12 for 9 h. Error bars represent SD across four biological replicates. (*E*) Western blot analysis of MMADHC in total lysate, cytosolic, and mitochondrial fractions of HEK293 cells transiently overexpressing MMADHC lacking the first 38 a.a. (MMADHCΔN38). TOM20 and α-Tubulin are used as controls for cell fractionation. Blot is representative of three biological replicates. (*F*) Western blot analysis of MMADHC and MTR in HEK293 cells stably overexpressing empty vector or MMADHCΔN38. Total protein is stained with Ponceau for loading control. Bar plots on the right show western blot quantification, error bars represent SD across five biological replicates. (*G*) MTR specific activity measured as in “*C*” in HEK293 cells transiently overexpressing empty vector or MMADHCΔN38. Error bars represent SD across three biological replicates.

To determine the role of MMADHC stabilized by depolarization, we performed TMT proteomics on control and MMADHC KO cells treated with or without anti+oligo. Of 7,376 proteins analyzed, MMADHC KO prevented the increase of only one protein following anti+oligo treatment: MTR (Fig. 3*A* and Dataset S2). No other proteins were differentially elevated or depleted by anti+oligo in control vs. MMADHC KO (Fig. 3*A*). These data allow us to draw a causal genetic link between the depolarization-induced increase in MTR and MMADHC, both of which were also hits in our initial screen (Fig. 1*A*) and conclude that the increase in MTR upon PMF collapse is MMADHC-dependent. Conversely, however, the increase in MMADHC upon PMF collapse is MTR-independent i.e. MMADHC increases comparably in response to anti+oligo in control and MTR KO cells (*SI Appendix*, Fig. S5*A*). Furthermore, MTR is attenuated in MMADHC KO even in the absence of anti+oligo (Fig. 3*B*), indicating that MMADHC influences basal levels of MTR. While MTR increases, the mitochondrial B_12_-dependent MMUT decreases upon PMF collapse in an MMADHC-independent manner in K562 and HEK293 cells (*SI Appendix*, Fig. S6 *A* and *B*).

Next, we examined the functional impact of mitochondrial depolarization-driven and MMADHC-dependent increase in MTR. We determined MTR activity in cell lysates in the presence of the MTR substrates, homocysteine and 5-methyl-tetrahydrofolate (THF), and a reactivation system (MTRR, NADPH, S-adenosylmethionine) (Fig. 3*C*, schematic). We observed a 1.5 to 2-fold increase in MTR specific activity in lysates of anti+oligo-treated cells, which was ablated by MMADHC KO in cells from two lineages, K562 and HEK293 (Fig. 3*C*). Western blot analysis of the same samples confirmed the MMADHC-dependent increase in MTR upon mitochondrial depolarization in both cell lines (*SI Appendix*, Fig. S5 *B* and *C*).

Additionally, we assayed MTR activity in live cells using deuterated formate to quantitatively trace the conversion of 5,10-methylene-THF to THF (Fig. 3*D*, schematic). In this experiment, we quantified: i) methionine labeling via the activities of MTHFR and MTR and ii) serine labeling via the activities of SHMT1/2 in K562 cells cultured in the presence of homocysteine to ensure it was not limiting for MTR activity. In addition, cells were cultured in the absence or presence of exogenous vitamin B_12_ to distinguish holo-MTR (that was already loaded with vitamin B_12_ in cells) from total MTR.

The deuterated formate tracing experiment revealed that homocysteine accumulates with anti+oligo treatment and is largely rescued by B_12_ supplementation (Fig. 3*D*). The presence of excess B_12_ in the culture medium is expected to support B_12_ loading independently of MMADHC, thus enabling assessment of total MTR activity. Concomitantly, deuterated methionine, which is indicative of MTR activity, increased in response to anti+oligo, independent of B_12_ supplementation (Fig. 3*D*) while the levels of unlabeled methionine were unchanged. B_12_ supplementation boosted deuterated methionine levels ~10-fold even without anti+oligo, while a smaller threefold increase was observed in the presence of anti+oligo (Fig. 3*D*). Finally, deuterated formate tracing to serine, depends on the conversion of 5,10-methylene-THF to THF via SHMT1/2and does not require B_12_. Anti+oligo increased unlabeled serine but decreased deuterated serine levels (*SI Appendix*, Fig. S7), suggesting SHMT1/2 reversal (16, 28). Collectively, the in vitro MTR activity and deuterated formate tracing reveal an increase in MTR activity upon mitochondrial depolarization and highlights that MTR exists largely in the apo form, limited by B_12_ loading.

To investigate if cytosolic stabilization of MMADHC in the absence of mitochondrial depolarization also increases MTR levels and activity, we characterized a ΔN38 variant of MMADHC that lacks the MTS. The longest predicted MTS for human MMADHC across TargetPv1.0 (29), v2.0, TPpred2 (30), and MitoFates (31) is 38 residues. Unlike the native protein, ΔN38-MMADHC was readily detected by western blot analysis even in the absence of anti+oligo and was localized almost exclusively in the cytosol (Fig. 3*E*). Overexpression of ΔN38-MMADHC increased MTR levels (Fig. 3*F*) and activity (Fig. 3*G*), demonstrating that cytosolic MMADHC is sufficient to boost both.

## Discussion

**Fig. 4.**
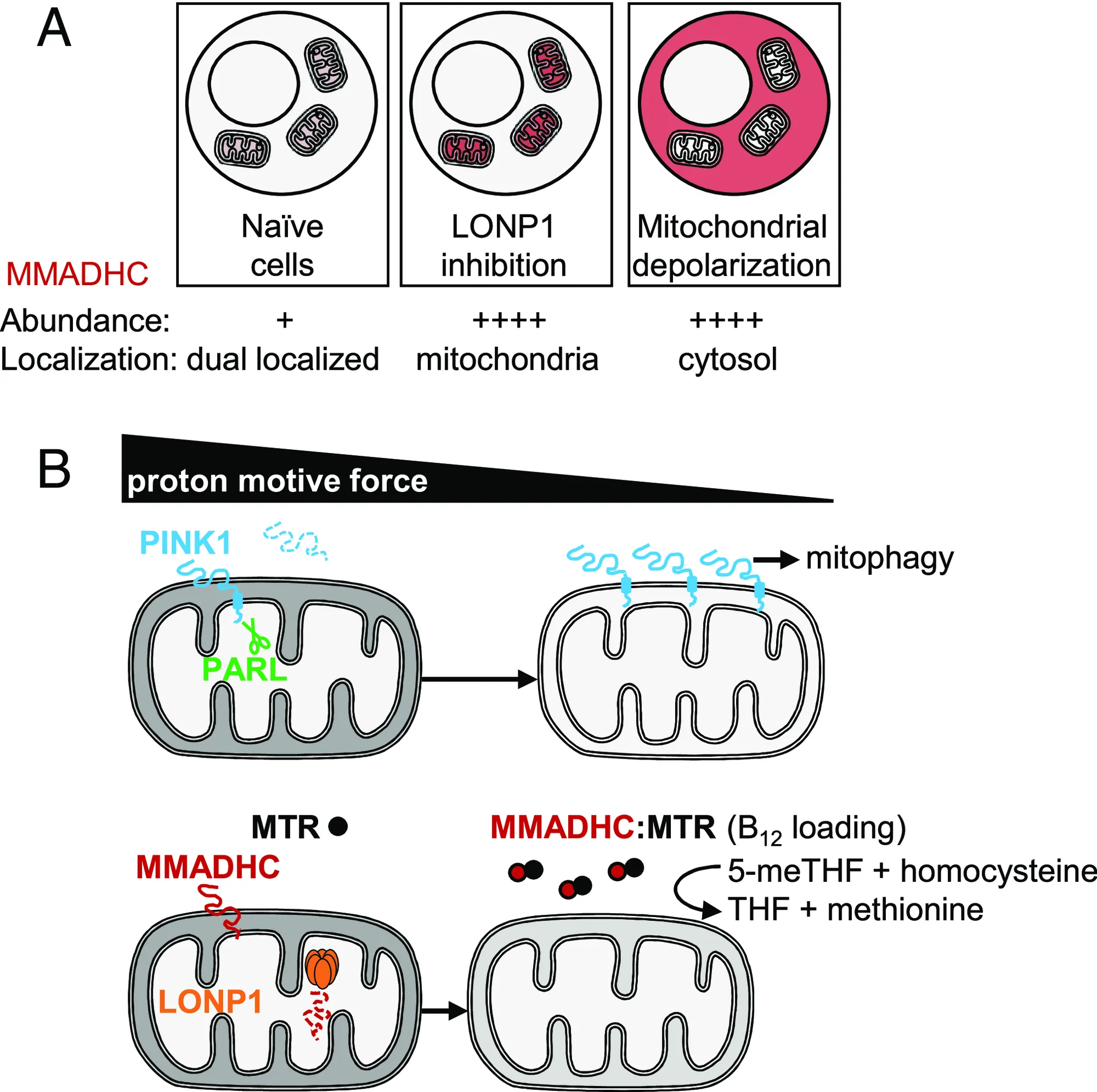
Model for the regulation of MMADHC by the mitochondrial PMF and LONP1. (*A*) Context-dependent MMADHC abundance and localization: MMADHC is present at very low steady state levels in cells with healthy mitochondria but accumulates in mitochondria with LONP1 inhibition and in the cytosol with mitochondrial depolarization. (*B*) MMADHC is more sensitive to collapse of the PMF than PINK1. Upon PMF collapse, MMADHC accumulates in the cytosol where it boosts the levels and activity of the B_12_-dependent MTR.

In this study, we sought to systematically discover proteins that, like PINK1 or ATFS-1, are stabilized upon mitochondrial PMF collapse. Our joint proteomic and RNA-seq screen in K562 cells identified 10 genes (six mitochondrial) that are up-regulated exclusively at the protein level in response to PMF collapse. We followed up on the top hit, the vitamin B_12_ chaperone MMADHC, and demonstrated that its short half-life is driven by its continual import into energized mitochondria and degradation by LONP1. We summarize our model for MMADHC regulation in Fig. 4. MMADHC can be conditionally stabilized in two cellular “zip-codes:” In mitochondria with LONP1 inhibition or in the cytosol with PMF collapse (Fig. 4*A*), where it increases the levels and activity of the cytosolic B_12_-dependent MTR (Fig. 4*B*). An open question is whether MMADHC stabilization and the resulting boost in cytosolic MTR activity is relevant in any specific physiological or disease state.

The two human B_12_-dependent enzymes reside in separate compartments (MMUT in mitochondria, MTR in the cytosol) with MMADHC being the only protein common to both branches after B_12_ is exported from the lysosome. Thus, regulating the distribution and stability of MMADHC likely allows cells to ration cofactor availability for each branch and to spatiotemporally compartmentalize vitamin B_12_ metabolism. In line with this, mutations in MMADHC that selectively impair the cytosolic or the mitochondrial B_12_ pathway, lead to a reciprocal increase in the B_12_ derivative used in the unaffected compartment, i.e., adenosylcobalamin or methylcobalamin, respectively (32). Retaining MMADHC in the cytosol during mitochondrial depolarization might be a mechanism for safeguarding the total intracellular B_12_ pool. Studies of fibroblasts from patients with inborn errors of cobalamin metabolism that are unable to enzymatically use B_12_, lose the cofactor to the extracellular medium (33). In murine hematopoietic stem cells (HSCs), mitochondrial turnover of Mmadhc by Afg3l2, the highest-expressed mitochondrial protease in HSCs, restricts B_12_-dependent TCA cycle anaplerosis and maintains HSC homeostasis (34). Further investigation is needed to systematically determine how different cell types/biological contexts influence MMADHC stability and localization to regulate B_12_ metabolism. For example, highly proliferative cells that rely on MTR to acquire the THF needed for nucleotide synthesis (35) may have higher levels of cytosolic MMADHC, while tissues such as liver, which has the highest expression of MMUT (GTEx Analysis Release V10, dbGaP Accession phs000424.v10.p2), may maintain relatively higher levels of mitochondrial MMADHC by favoring its import or potentially slowing its degradation.

The continuous mitochondrial turnover and conditional cytosolic retention of MMADHC upon PMF collapse allows cells to couple increased MTR activity to PMF collapse. We note that the immediate metabolic “neighborhood” of MTR includes pathways already known to be transcriptionally elevated by ETC dysfunction via the ATF4 response, namely serine synthesis, transsulfuration, and xCT. We now report a distinct, ATF4-independent, and posttranscriptional mechanism of increasing MMADHC, and consequently MTR, specifically upon PMF collapse but not with other ETC inhibition. We speculate that increased MTR is needed upon PMF collapse because of defective homocysteine clearance and SHMT1/2 reversal revealed by our formate tracing experiment. The latter is consistent with our previous study which identified serine as the top increasing metabolite in mtDNA-depleted cells and SHMT2 reversal upon ETC inhibition (18).

Cells exploit the conditional stabilization and localization of mitochondrial proteins not only for signaling stress, as in the case of PINK1, ATFS-1, and DELE1, but also for modulating metabolism. Besides MMADHC, our screen also recovered STAR, the steroidogenic acute regulatory protein, which functions at the OMM to facilitate the rate-limiting import of cholesterol to the IMM (36). Like MMADHC, STAR is also rapidly degraded by LONP1 (37) in the mitochondrial matrix and this turnover is thought to be crucial for regulating steroid synthesis. In uricotelic (uric acid-excreting) vertebrates like chickens, another metabolic enzyme is differentially mitochondria-localized: glutamine synthetase (GS) is cytosolic in astrocytes but mitochondrial in hepatocytes where it is critical for detoxifying ammonia. This differential localization is achieved by GS harboring a weak MTS and hepatocyte mitochondria specifically having a high enough PMF to drive GS import (38).

The “importability” of mitochondrial proteins, which is dictated by intrinsic protein features such as MTS strength and the bioenergetic state, appears to be finely tuned for each protein’s function and organelle homeostasis. We observed that PINK1 is more resilient to PMF collapse than MMADHC, likely due to PINK1 having a stronger MTS, which allows cells to reserve PINK1-dependent mitophagy only for severe mitochondrial depolarization. A comparative study of MTS strengths in yeast uncovered remarkable variability in the import kinetics of proteins not known to be involved in stress signaling (39). We therefore speculate many additional mitochondrial proteins than currently appreciated to be stabilized/(re)localized at specific PMF thresholds.

## Materials and Methods

### Cell Culture.

K562 (female), HEK293 (female), and HT29 (female) cells were obtained from the ATCC and cultured in DMEM (Gibco 11995-065) with 10% FBS (Sigma Aldrich F2442) and 100 U/mL penicillin/ streptomycin at 5% CO_2_ and 37 °C. For experiments with ETC inhibitors and uncouplers, cells were supplemented with 50 μg/mL uridine. When necessary, K562 cells were selected with 2 μg/mL puromycin (GIBCO). Cell lines were authenticated by STR profiling (ATCC) and tested to ensure the absence of *Mycoplasma* once every 3 mo. For experiments with CRISPR-Cas9 KO, polyclonal cells were used throughout.

### TMT Proteomics.

Quantitative proteomics was performed at the Thermo Fisher Scientific Center for Multiplexed Proteomics (Harvard).

#### Sample preparation.

Cell pellets were lysed in 8 M urea, 200 mM EPPS pH 8.5, protease and phosphatase inhibitors. For each sample, 150 μg protein was reduced with TCEP, alkylated with iodoacetamide, and further reduced with dithiothreitol (DTT). Proteins were precipitated onto SP3 beads and buffer exchanged for digestion with Lys-C (1:50) overnight at room temperature and trypsin (1:50) for 6 h at 37 °C. Peptides were labeled with TMTPro reagents. Complete TMT labeling was confirmed by running a ratio check on a pooled sample comprising 2 μL of each sample. All 18 TMTPro-labeled samples were pooled and desalted by Sep-pak. Phosphopeptides were enriched using a Peirce High-Select Fe-NTA Phosphopeptide Enrichment Kit and the flow through was fractionated into 24 fractions using basic reverse-phase HPLC and used for total proteome profiling. Fractions were solubilized, desalted by stage tip, and analyzed on an Orbitrap Lumos mass spectrometer (total proteome) or an Orbitrap Eclipse mass spectrometer with FAIMS enabled (phospho-proteome). Phosphopeptides were shot twice using different FAIMS CVs.

#### Data analysis.

MS2 spectra were searched using the COMET algorithm against a Human Uniprot composite database containing its reverse complement and known contaminants. For phospho-proteome, a database with phosphorylation on Ser, Thr, or Tyr as differential modifications was searched. For peptide matches in the total proteome, a 1% false discovery rate (FDR) was set using the target-decoy strategy and linear discriminant analysis. Proteins were quantified only from peptides with a summed SN threshold of >180. Phosphopeptide FDR was set to 1% by LDA analysis of modified peptides only. Phosphosite localization was scored by ModScore. 18-plex Comet Search Parameters: Peptide Mass Tolerance: 50 ppm, Fragment Ion Tolerance: 0.4 ppm (0.02 ppm for phosphopeptides), Max Internal Cleavage Site: 2 (3 for phosphopeptides), Max differential/Sites: 5. Methionine oxidation and Phosphorylation at STY were used as variable modification. Localized sites have A-Score >13 with 95% confidence of detection. 18-plex TMTpro Reporter Quant Parameter: Proteome (MS3)–tolerance = 0.003, ms2_isolation_width = 0.7, ms3_isolation_width = 1.2. Phos (MS2)–tolerance = 0.003, ms2_isolation_width = 0.5. Proteins quantified by >1 peptide were analyzed.

### RNA Sequencing.

Library preparation and sequencing was conducted by Azenta Life Sciences (South Plainfield, NJ).

#### Library preparation with PolyA selection and illumina sequencing.

RNA was quantified using Qubit 2.0 Fluorometer (ThermoFisher Scientific) and RNA integrity was verified with 4200 TapeStation (Agilent Technologies). Strand-specific RNA sequencing libraries were prepared using NEBNext Ultra II Directional RNA Library Prep Kit for Illumina as per the manufacturer’s instructions (NEB). Briefly, the enriched RNAs were fragmented for 8 min at 94 °C. Random hexamers were used to prime first and second strand cDNA synthesis. The second strand cDNA was marked by incorporating dUTP to preserve strand specificity. cDNAs were adenylated at 3′ ends and ligated to indexed adapters. The library was enriched by limited cycle PCR, validated on the Agilent TapeStation (Agilent Technologies), and quantified using Qubit 2.0 Fluorometer (ThermoFisher Scientific) and qPCR (KAPA Biosystems). Multiplexed sequencing libraries were sequenced on the Illumina NovaSeq X Plus in a 2 × 150 bp Paired End (PE) configuration. Image analysis and base calls were made by the NovaSeq Control Software (NCS). Raw sequence data (.bcl files) were converted to fastq files and demultiplexed using Illumina bcl2fastq 2.20, allowing one mismatch in the index sequence.

#### Data analysis.

Sequencing reads were trimmed to remove any adapter sequences and nucleotides with poor quality, then mapped to the reference genome using STAR aligner v.2.5.2b, which accounts for splice junctions. The resulting BAM files were used to count reads uniquely falling within exon junctions. All reads mapping to a gene were normalized to a “Transcripts Per Million” (TPM) metric of abundance. Fold change was calculated as (average of the RNA abundance across biological replicates of cells treated with anti+oligo for 3 d + 0.1)/(average of the RNA abundance across biological replicates of cells treated with DMSO for 3 d + 0.1).

### Western Blot.

Equal number of cells from each condition (1 to 2 × 10^6^) were harvested by pelleting at 650 g for 2 min, washed once with PBS, and lysed directly in 150 μL LDS sample buffer (Nupage NP0007) diluted to 2× with water or 100 μL lysis buffer containing 25 mM HEPES, pH7.5, 25 mM KCl, 0.5% NONIDET P40, 1% (v/v) protease inhibitor cocktail (Sigma Cat#P8340) and 1 mM PMSF. Ten percent of the sample (or 40 to 80 μg protein) was run on Novex™ 4 to 20% Tris-Glycine Mini Gels (Thermo Fisher Scientific XP04200BOX) or polyacrylamide gels (15% for Tom20, 12% for Tubulin and MMADHC, 6% for MTR) and transferred to a 0.2 μm PVDF membrane (BioRad 1704156). Then, the gel was stained with Coomassie and membrane with Ponceau at the end of the western blot, where shown, to stain total proteins. Membranes were blocked for 10 min in 5% nonfat dry milk prepared in TBST. Membranes were then incubated with primary antibody diluted (1:5,000 for MMADHC polyclonal, 1:1,000 for MMADHC monoclonal, 1:10,000 tubulin and TOM20, 1:1,000 for all others) in 5% milk overnight at 4 °C. The following primary antibodies were used:

**Table:** 

Mouse monoclonal anti-MMADHC	Origene	Cat#TA800573; RRID:AB_2625545
Rabbit polyclonal anti-MMADHC	Abcam	Cat#ab204313 RRID:AB_3696890
Rabbit monoclonal anti-PINK1	Abcam	Cat#ab216144 RRID: AB_2927726
Rabbit monoclonal anti-phospho-S65 ubiquitin	Cell signaling	Cat#62802S RRID:RRID:AB_2799632
Rabbit monoclonal anti-ATF4	Cell Signaling	Cat#11815S RRID:AB_2616025
Rabbit monoclonal anti-Methionine synthase	Cell Signaling	Cat#68796S RRID:AB_2799753
Mouse monoclonal anti-Tom20	Sigma-Aldrich	Cat#MABT166 RRID:AB_2890986
Mouse monoclonal anti-alpha-Tubulin	Abcam	Cat#ab7291 RRID:AB_2241126

Membranes were then washed in TBST 4 times for 5 min each at room temperature. Next, they were incubated in HRP-linked α-rabbit (Cell Signaling 7074P2 1:5,000 or Abcam, ab205719, 1:20,000) or α-mouse (Cytiva NA931V 1:5,000, or Kindle Biosciences, R1005, 1:1,000) secondary antibodies diluted 1:5,000 in 5% nonfat dry milk, for 1 h at room temperature. Membranes were washed again in TBST as before, incubated with 2 mL of Lightning® Plus ECL (Revvity NEL103E001EA) or Kwikquant Western Blot Detection kit, Kindle Biosciences (Cat#R1004), and exposed to Amersham Hyperfilm™ (GE Healthcare) or and images were collected with a KwikQuant Imager (Kindle Biosciences). Band intensities were quantified using GelQuant (BiochemLabSolutions) or ImageJ and normalized to total protein.

### Quantitative PCR.

RNA was purified using the RNeasy kit (QIAGEN) with on-column DNase (Qiagen ID. 79254) treatment per the manufacturer’s instructions. RNA was quantified using a nanodrop and 1 μg was converted to cDNA using the High-Capacity RNA-to-cDNA™ Kit (Applied Biosystems Cat#4387406). QPCR was performed using the primers listed in the Key Resources Table and iQ™ SYBR® Green Supermix (Bio-rad). Fold change was calculated using the ΔΔCt method, normalizing genes of interest to ACTB. Primers used:

HSPD1_F: ATGATGCTATGGCTGGAGATT, R: CACCAGCAGCATCCAATAAAG
ACTB_F: CACAGAGCCTCGCCTTT, R: GCGGCGATATCATCATCCAT

### Cell Fractionation.

0.8 to 1 × 10^8^ confluent HEK293 or HT29 cells were washed with PBS and pelleted at 700×*g* for 5 min. The cells were homogenized in the homogenization buffer containing 20 mM HEPES, pH7.6, 220 mM mannitol, 70 mM sucrose, 1 mM EDTA + 0.5 mM PMSF. The cell debris was removed by centrifugation at 1,000×*g* for 7 min at 4 °C. The crude mitochondria were separated from the cell lysates by centrifugation at 10,000×*g* for 15 min and washed once with the homogenization buffer. The isolated cytoplasmic and mitochondrial fractions were frozen in liquid nitrogen and stored at −80 °C until further use.

### Target Guide Cloning.

CRISPR guide sequences used were

MMADHC sgRNA #1 TAACTAAAGAGCAAAATCCT
MMADHC sgRNA #2 TGAAAGACATGAGTTTGTGA
MTR sgRNA #1 CACCTGCATTGGGATAACAG
MTR sgRNA #2 GTGTACGGTCCAATCCTGAA
sgOR7G3 sgRNA GGTGAAACAGATGTCGACCA

Guides were cloned according to the protocol available on addgene with pLentiCRISPRv2. Briefly, 5 μg of the vector was digested with FastDigest BsmBI (Fermentas) and dephosphorylated with FastAP (Fermentas) in the FastDigest buffer for 30 min at 37 °C. The larger fragment was gel purified using the QIAquick Gel Extraction Kit. Oligos for the guides listed in the key resources table were phosphorylated with T4 PNK for 30 min at 37 °C and annealed by heating to 95 °C 5 min and then ramping down to 25 °C at 5 °C/min. The phosphorylated and annealed oligos were diluted 1:200 and ligated to the BsmBI-digested plasmid with Quick Ligase (NEB M2200S) at room temperature for 10 min. The ligation was transformed into Stbl3 bacteria and confirmed by Sanger sequencing.

### Lentivirus Production.

1 × 10^6^ HEK293T cells were seeded in 2 mL medium/well in a six-well plate (three wells per lentivirus). The next day, each well was transfected with 500 μL of transfection mixture. This mixture contained 6 μL Lipofectamine 2000 (Thermo Fisher Scientific Cat#11668019), 1 μg of the lentiviral vector of interest, 900 ng psPAX2 (Addgene 12260), and 100 ng pMD2.G (Addgene 12259) all diluted in 500 μL Opti-MEM medium (Gibco Cat#31985062) and incubated at room temperature for 20 min before adding to cells. 2 d after transfection, media were harvested and spun at 650×*g* for 5 min to pellet and eliminate floating cells. The media containing lentivirus were either aliquoted and stored at −80 °C or directly used for lentiviral infection.

### KO Generation.

Lentivirus was added with 8 μg/mL polybrene to 2 million K562 (or 1 million HEK293) cells in 2 mL medium in one well of a six-well plate. The plate was spun at 500 g for 30 min (or 1,200×*g* for 45 min) at room temperature. The next day, infected cells were expanded to a larger culture volume and selected with 2 μg/mL puromycin on day 2 to 3. KO was confirmed by western blot after treating cells with or without 100 nM antimycin and 30 nM oligomycin, which allows detection of MMADHC in control cells.

### Stable Overexpression.

The following primers were used to generate the ΔN38 MMADHC construct from the C2ORF25/MMADHC (NM_015702) Human Tagged ORF Clone (OrigeneRC204801)

F: ATCTGCCGCCGCGATCGCCATGTCGGATGAGTCTCATGTG
R: ATCGCGGCGGCAGAT

0.5 × 10^6^ HEK293 cells were seeded in 2 mL medium/well in a six-well plate (three wells per lentivirus). The next day, each well was transfected with 250 μL of the transfection mixture in 2 mL medium. This mixture contained 3.75 μL Lipofectamine 3000 (Invitrogen Cat#L3000008), 2.5 μg of the vector of interest, 5 μL P3000, all diluted in 250 μL Opti-MEM medium (Gibco Cat#31985062) and incubated at room temperature for 20 min before adding to cells. 2 d after transfection, the transfected cells were expanded to a larger culture volume and selected with 100 μg/mL Geneticin (Gibco, Cat#10131035).

### MTR Activity Assay in Cell Lysates.

For preparing cell lysate, ~500 µg of cell pellet was resuspended in 1mL of lysis buffer [10 mM Tris-HCl pH–7.4, 100 mM NaCl, 2 mM ethylenediaminetetraacetic acid (EDTA), 1% Nonidet P-40 (NP-40), 1 mM DTT, and 1 mM phenylmethylsulfonyl fluoride (PMSF)]. 25 µM methylcobalamin (MeCbl) was added to the cell lysate and incubated in the dark on ice for 10 min, with gentle mixing every 3 min. The cell lysate was centrifuged at 13,000 rpm for 15 min to pellet cell debris and the supernatant was used to measure MTR activity. MTR activity was measured using the nonradioactive assay as described previously (1). The reaction mixture (800 µL total volume) containing 1 mM D,L-homocysteine, 150 µM S-adenosylmethionine (AdoMet), 1.2 µM methionine synthase reductase (MTRR) and 250 µL cell lysate (equivalent to 3 mg of total protein based on Bradford based quantification) in 100 mM potassium phosphate buffer, pH 7.4 was incubated at 37 °C for 5 min following which 300 µM 5-methyltetrahydrofolate [(*6S*)-CH_3_-H_4_F] was added and incubated at 37 °C for an additional 2 min. The reaction was initiated by addition of 200 µM NADPH. Aliquots (150 µL) of the reaction mixture were removed at the desired times (typically 5 min, 10 min, 15 min, 20 min, and 25 min) and quenched by adding 37.5 µL of a 1:1 mixture of formic acid:HCl. The reaction was heated at 80 °C for 10 min and cooled, which led to methenyl-H_4_F formation. The reaction was centrifuged at 13,000 rpm for 7 min and the supernatant was used to determine the concentration of methenyl-H_4_F at 350 nm using an extinction coefficient of 26.5 × 10^3^ M^−1^ cm^−1^. For each reaction, the initial velocity (V) was obtained from the slope of the line fitted to the product formed vs. time plot using the equation:

[1] [P]=V∗t,

where [*P*] is the concentration of product formed, and *t* is the time of the reaction.

### Deuterated Formate Tracing.

K562 cells were treated with DMSO or 100 nM Antimycin + 30 nM Oligomycin for 3 d and grown in DMEM (Gibco 11995-065) with 10% FBS (Sigma-Aldrich Cat#F2442), 100 U/mL penicillin/streptomycin, and 50 μg/mL uridine. 9 h prior to harvesting for metabolomics, 1 million cells were washed with, and resuspended in, 2 mL DMEM with 10% dialyzed FBS (Gibco Cat#26400044), 100 U/mL penicillin/streptomycin, 50 μg/mL uridine. Each sample was first split into two groups, receiving 3 mM Sodium formate that was either fully deuterated (Cambridge Isotope Laboratories, Cat#DLM-1361-5) or undeuterated (Sigma-Aldrich, Cat#247596-100G). Each formate group was further split into two groups: one received supplemental 100 μM homocystine (Sigma-Aldrich H6010) + 100 μM cyanocobalamin (Sigma-Aldrich V6629) and another with only supplemental 100 μM homocystine but no B_12_ for the duration of labeling (9 h). To harvest, cells were pelleted at 650×*g* for 2 min, washed with 1 mL PBS, and spun for 650×*g* 2 min. PBS was aspirated as close to the cell pellet as possible and tubes were placed on ice immediately. 400 μL of prechilled 4:4:2 methanol/acetonitrile/water + 0.1 M formic acid was added to pellets, tubes were vortexed for 10 s, and kept on ice for 3 min. Samples were neutralized with 35 μL 15% ammonium bicarbonate, vortexed, and stored at −80°C for mass spectrometry analysis. 5 μL of extracted samples analyzed using liquid chromatography coupled with a mass spectrometer (LC–MS). The LC separation was performed on a Waters XBridge BEH Amide column (2.1 mm × 150 mm × 2.5 µm) with column temperature at 25 °C. Buffer A was 5% acetonitrile with 20 mM ammonium acetate, 0.25% ammonium hydroxide, pH 9.0, and buffer B was 100% acetonitrile. The total flow rate was set to 0.15 mL/min and the samples were loaded at 90% B. The gradient was, 0 min, 90% B; 2 min, 90% B; 3 min, 75%; 7 min, 75% B; 8 min, 70%, 9 min, 70% B; 10 min, 50% B; 12 min, 50% B; 13 min, 25% B; 14 min, 25% B; 16 min, 0% B, 20.5 min, 0% B; 21 min, 90% B; 25 min, 90% B. The total running time was 25 min. The eluate was directed into Q-Exactive Plus Orbitrap mass spectrometer (Thermo Fisher Scientific, Waltham, MA) with HESI probe operating in polarity switching mode. MS parameters were sheath gas flow 50, aux gas flow 10, sweep gas flow 2, spray voltage 3.3 kV in both negative and positive modes, capillary temperature 310 °C, S-lens RF level 50 and aux gas heater temperature 370 °C. Other MS parameters included resolution of 140,000 at m/z 200, automatic gain control (AGC) target at 3e6, maximum injection time of 200 ms and scan range of m/z 120 to 1,000 in the positive mode and m/z 70 to 1000 in the negative mode respectively. Data were acquired using Xcalibur (v.4.1.31.9, Thermo Fisher). All LC–MS data were collected with samples injected in a randomized order with pooled quality control samples interspersed throughout the runs to quantify instrument performance during the run. Data were analyzed via MAVEN software using an in-house library (40, 41) with 5 ppm mass tolerance and isotope labeling data were corrected for natural isotope abundance (42).

## Supplementary Material

Appendix 01 (PDF)

Dataset S01 (XLSX)

Dataset S02 (XLSX)

## Data Availability

The mass spectrometry proteomics data have been deposited to the ProteomeXchange Consortium via the PRIDE partner repository with the dataset identifier PXD079746 (43). The RNA sequencing data are available through the GEO repository via accession number GSE335487 (44). This paper does not report original code. All other data are included in the manuscript and/or supporting information.
