# Supplementary material for: Mitochondrial depolarization stabilizes the vitamin B12 chaperone MMADHC in the cytosol to increase MTR activity
Source: Proc Natl Acad Sci U S A. 2026 Sep 8;123(37):e2537797123. doi: 10.1073/pnas.2537797123 (PMC13578772; doi:10.1073/pnas.2537797123)
Supplement: Supplementary file 1 [file pnas.2537797123.sapp.pdf]

PNAS

Supporting Information for

Mitochondrial depolarization stabilizes the vitamin B12 chaperone MMADHC in the cytosol to increase MTR activity

Sneha P. Rath[1,2,3,*], Zhu Li[4,*], Arkajit Guha[4], Fangcong Dong[1,2,3], Ruma Banerjee[4,**] and Vamsi K. Mootha[1,2,3,5**]

1. Department of Molecular Biology, Mass General Hospital, Boston, MA 02114, USA.
2. Department of Systems Biology and Medicine, Harvard Medical School, Boston, MA 02115, USA.
3. Broad Institute of MIT and Harvard, Cambridge, MA 02142, USA.
4. Department of Biological Chemistry, University of Michigan, Ann Arbor, MI 48109, USA.
5. Howard Hughes Medical Institute, Boston, MA 02114, USA.

*Contributed equally

****Co-corresponding authors:** Vamsi K. Mootha and Ruma Banerjee
Email(s): vamsi@hms.harvard.edu and rbanerje@umich.edu

This PDF file includes:

Figures S1 to S7 with figure legends
Legends for Datasets S1-S2

Other supporting materials for this manuscript include the following:

Datasets S1, S2

Figures

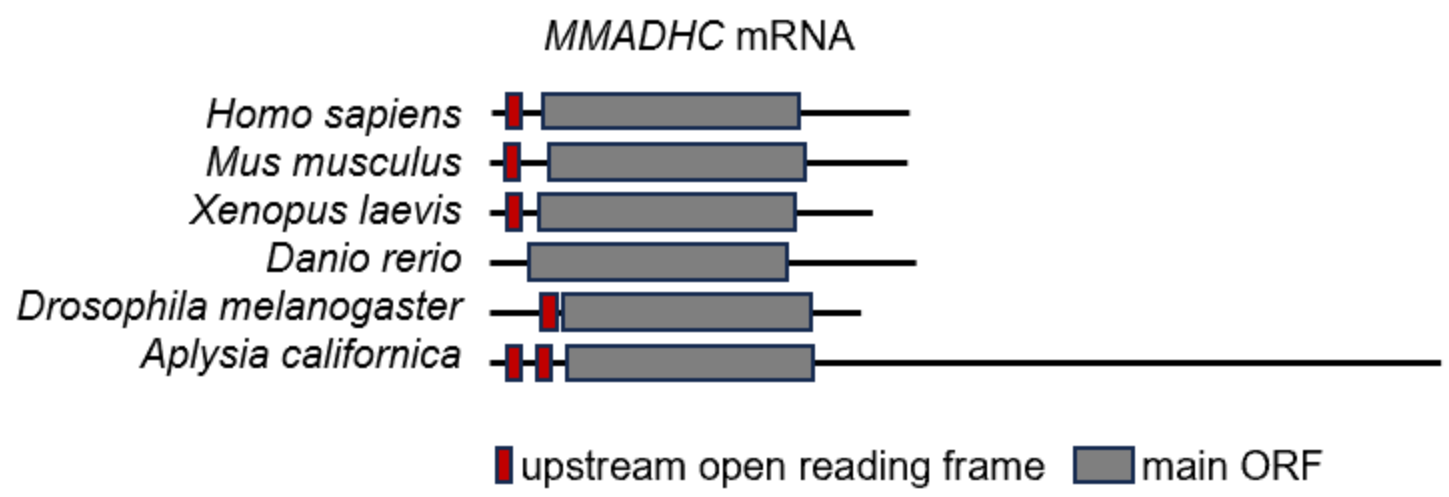

Fig. S1. Upstream open reading frames in MMADHC mRNA, related to Figure 1. Schematic of upstream open reading frames (uORFs) in *MMADHC* mRNA in the indicated species. The mRNA and main ORF lengths are drawn to scale, red boxes indicate presence of uORF.

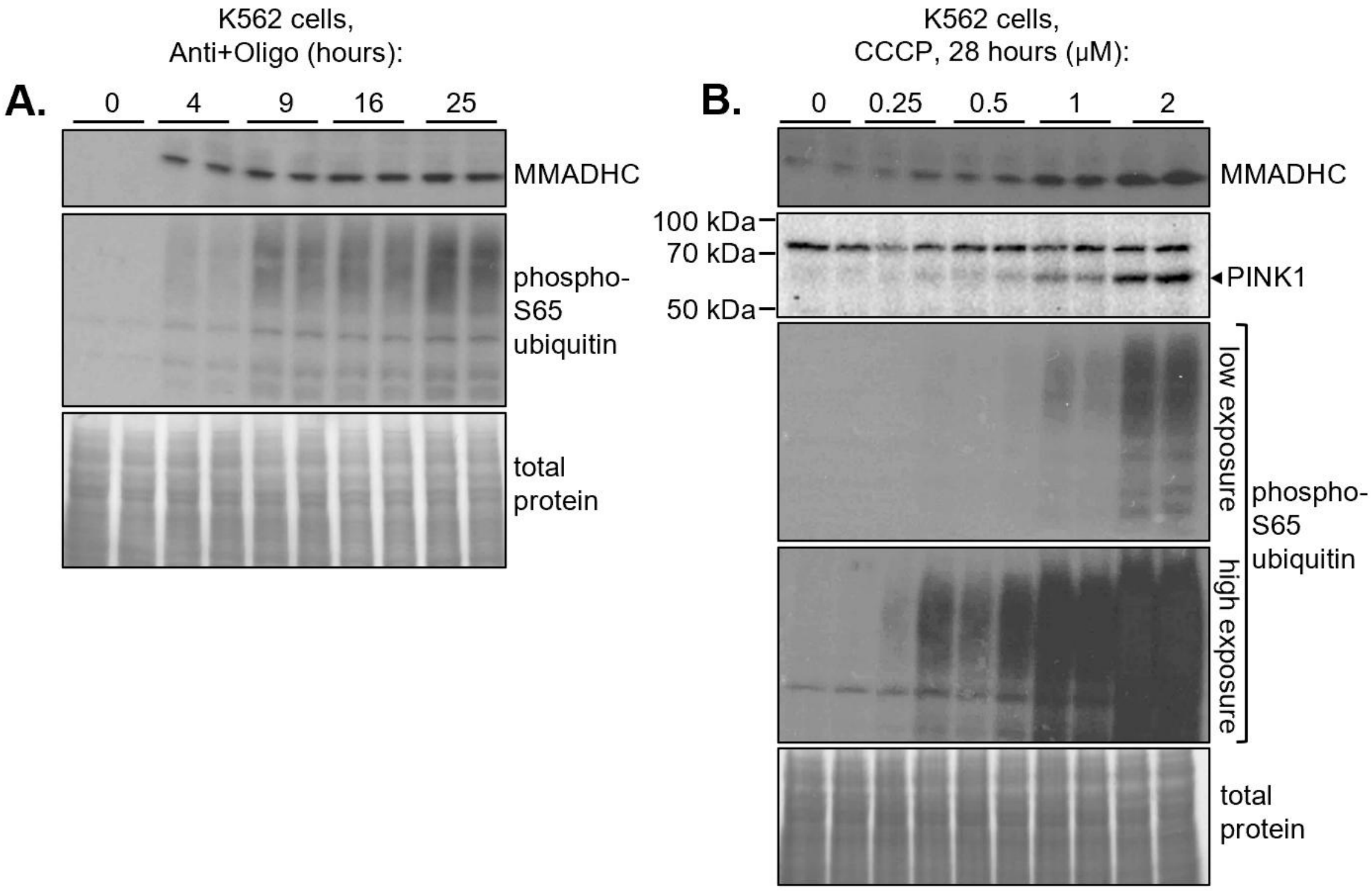

Fig. S2. Comparison of the increases in MMADHC vs PINK1-dependent phospho-S65 ubiquitin upon varying durations and strengths of mitochondrial depolarization, related to Figure 2.

(A) Western blot analysis of MMADHC and phospho-S65 ubiquitin in K562 cells treated with antimycin and oligomycin for the indicated durations. Total protein is stained with Coomassie for loading control.

(B) Western blot analysis of MMADHC, PINK1 (correct band indicated by black arrowhead), and PINK1 substrate phospho-S65 ubiquitin in K562 cells treated with the indicated doses of CCCP for 28 hours. Multiple exposures are shown for phospho-S65 ubiquitin. Total protein is stained with Coomassie for loading control.

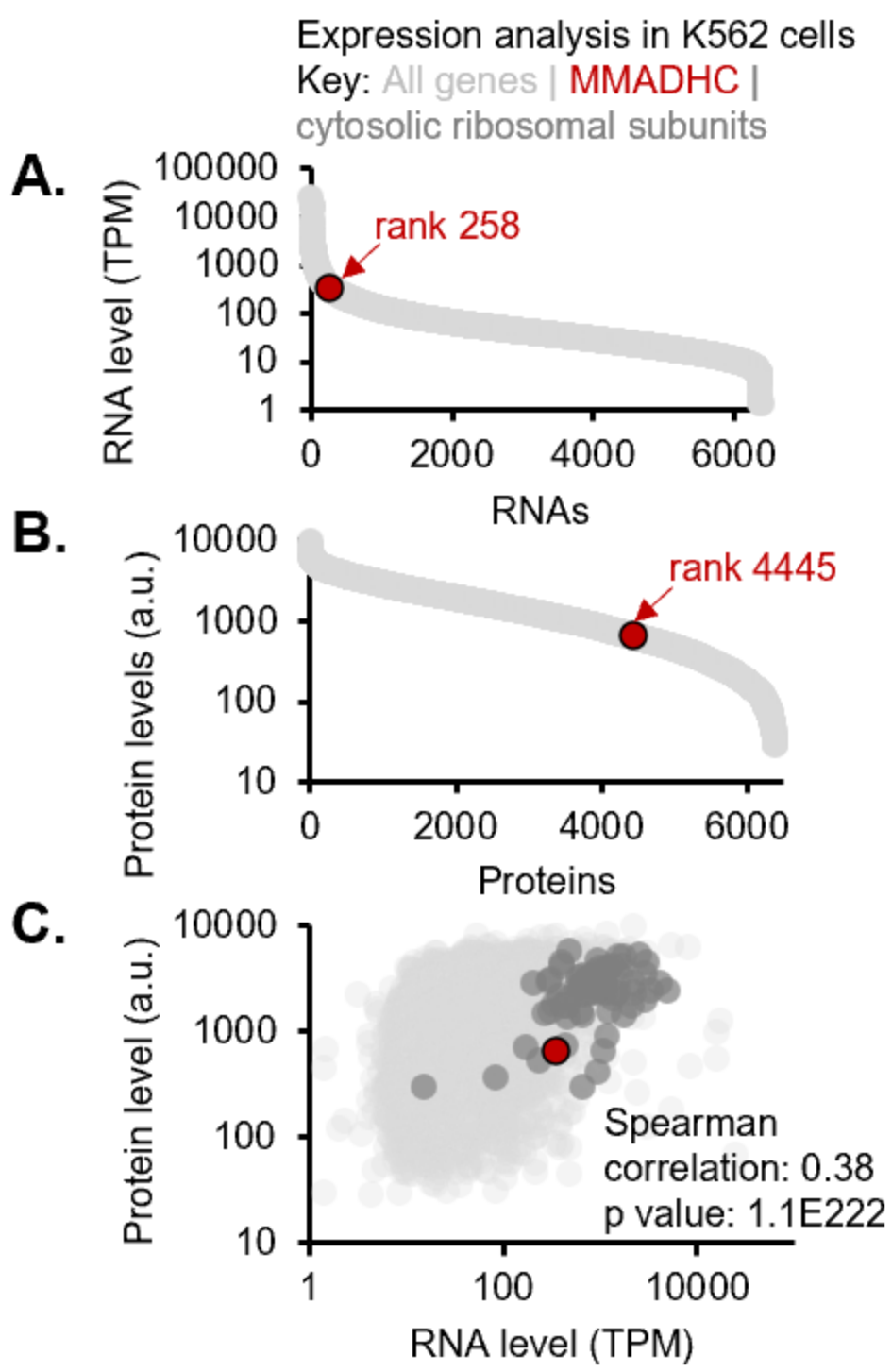

Fig. S3. MMADHC protein, but not RNA, is lowly expressed, related to Figure 2.
(A) Genes ranked by RNA level in naïve K562 cells measured by RNAseq.
(B) Genes ranked by protein level in naïve K562 cells measured by TMT proteomics.
(C) Scatter plot of protein vs RNA level. Red point corresponds to MMADHC, dark grey points correspond to cytosolic ribosomal proteins.

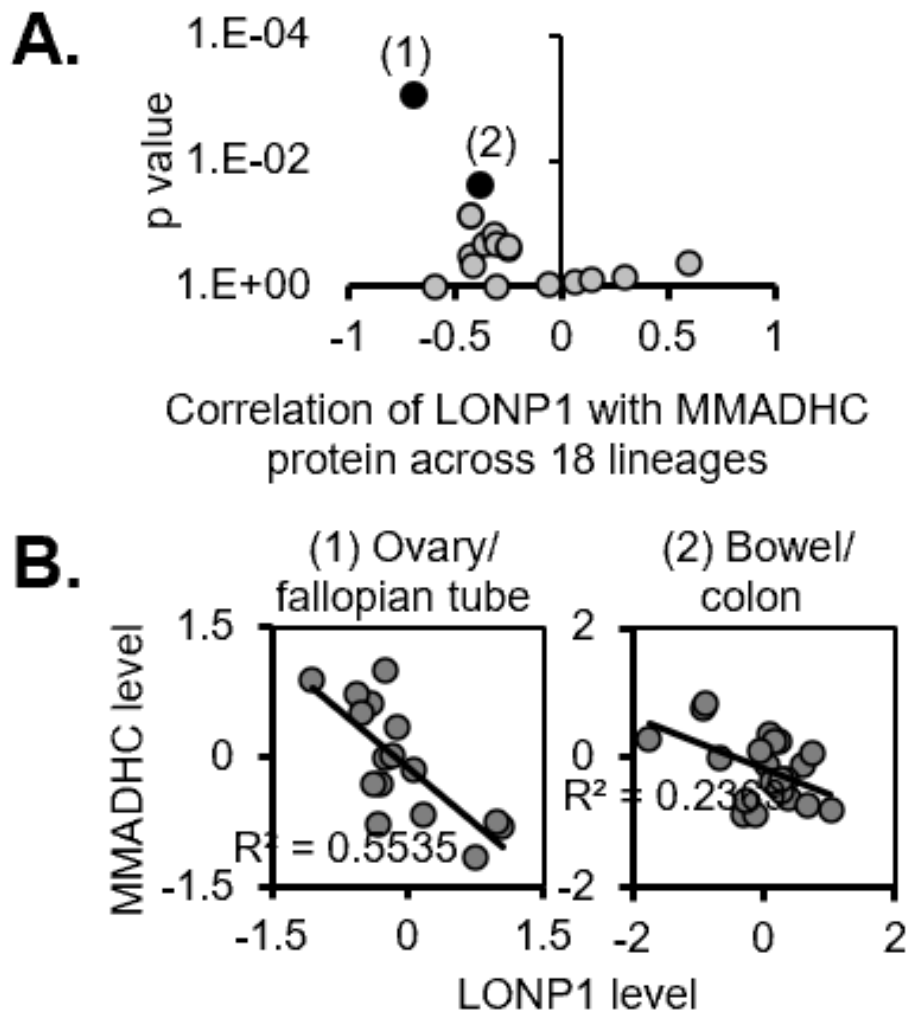

Fig. S4. Correlation of MMADHC and LONP1 across CCLE by lineage, related to Figure 2.
(A) Volcano plot of correlation between MMADHC and LONP1 in each CCLE lineage.
(B) Scatter plots of MMADHC vs LONP1 protein levels in the top two lineages in which the two proteins are most significantly negatively correlated.

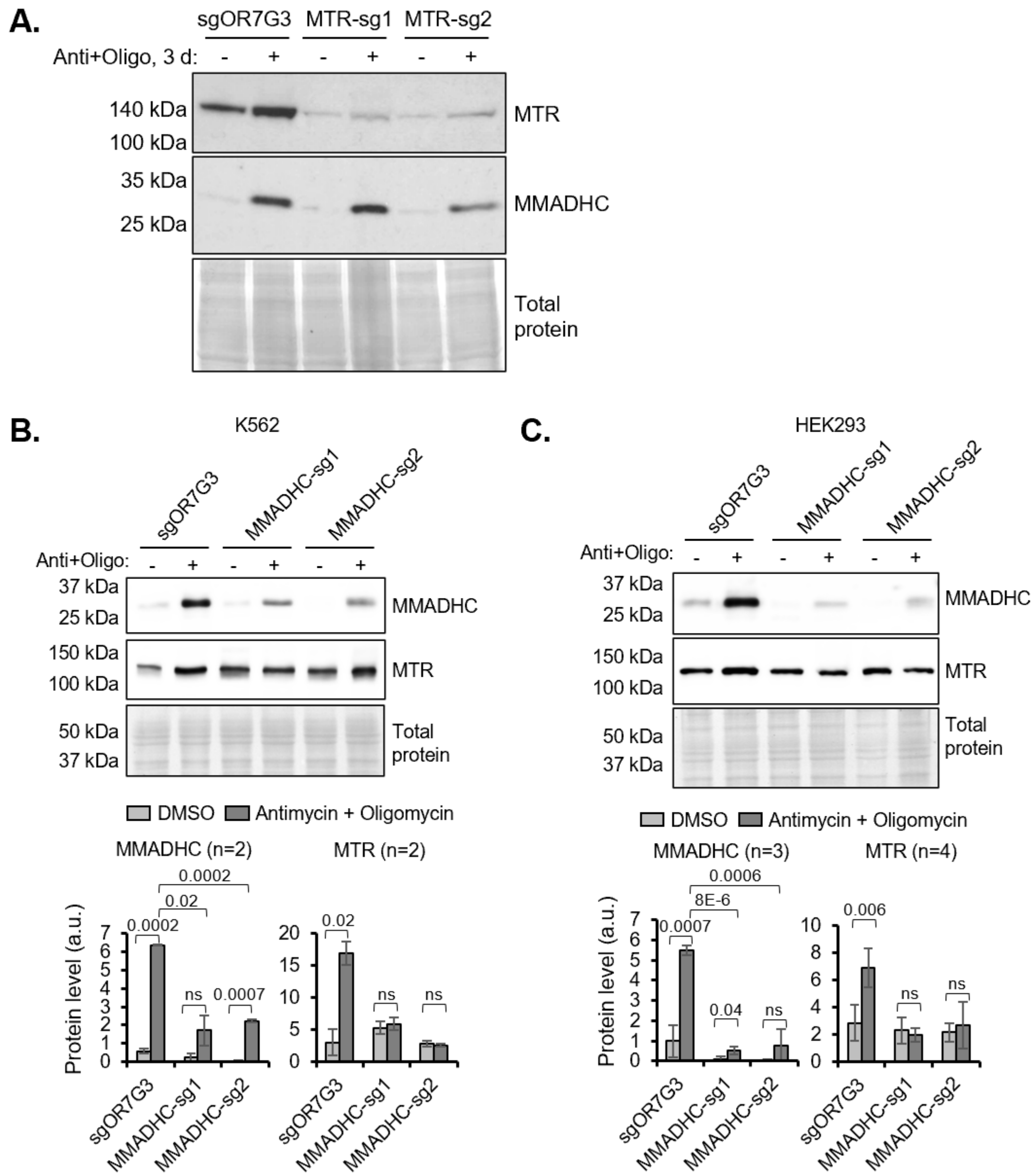

Fig. S5. Increase of MTR upon mitochondrial depolarization is MMADHC-dependent and not vice versa, related to Figure 3.

(A) Western blot analysis of MMADHC and MTR in cutting control (sgOR7G3) and MTR KO K562 cells treated with antimycin and oligomycin for 3 days.

(B, C) Western blot analysis of MMADHC and MTR in cutting control (sgOR7G3) and MMADHC KO K562 **(B)** and HEK293 **(C)** cells treated with antimycin and oligomycin for 3 days. Bar plots show quantification of western blots normalized to total protein. Error bars show standard deviation across biological replicates indicated above each plot.

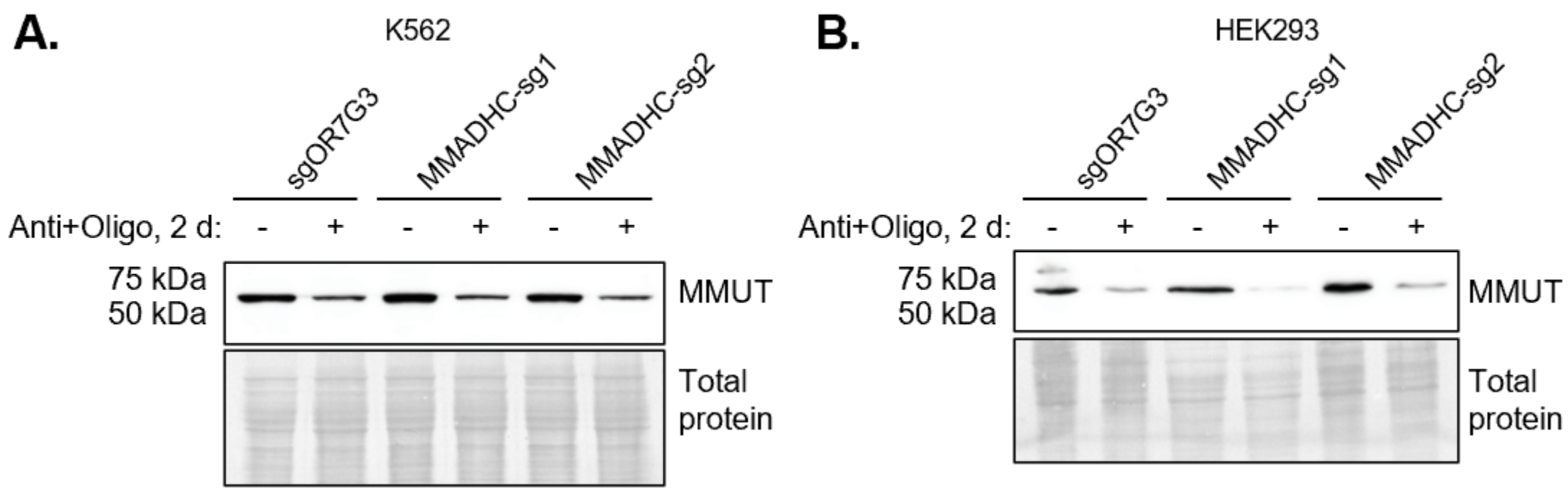

Fig S6. Decrease in MMUT upon mitochondrial depolarization, related to Figure 3.
(A, B) Western blot analysis of MMUT in cutting control (sgOR7G3) and MMADHC KO K562 **(A)** and HEK293 **(B)** cells treated with antimycin and oligomycin for two days.

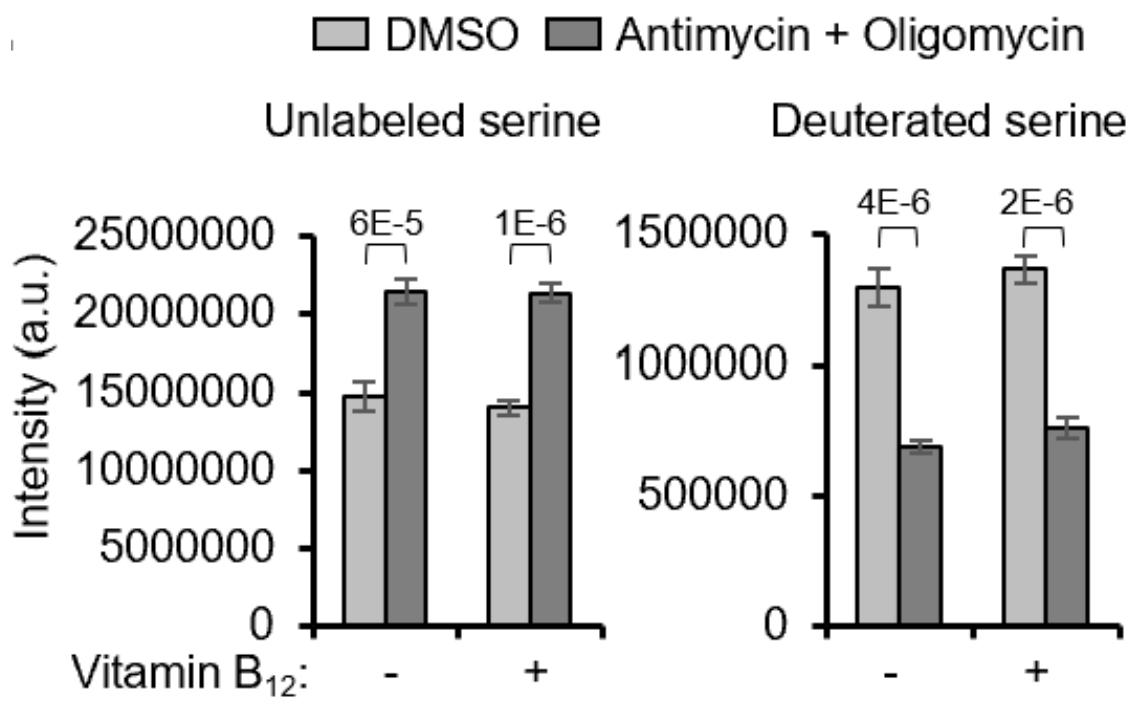

Fig. S7. Formate-to-serine flux decreases in cells treated with antimycin and oligomycin, related to Figure 3.
Levels of undeuterated and deuterated serine quantified by mass spectrometry in K562 cells treated with antimycin and oligomycin for 3 days and labelled with fully deuterated formate for 9 hours in the presence or absence of vitamin B_{12}. Error bars represent standard deviation of four biological replicates.

Dataset S1 (separate file). RNAseq and proteomics data related to main figure 1A: RNAseq and TMT proteomics of sgOR7G3 (CRISPR cutting control) K562 cells treated with or without antimycin+oligomycin to depolarize mitochondria for three days,

Dataset S2 (separate file). Proteomics data related to main figure 3A: TMT proteomics of sgOR7G3 (CRISPR cutting control) and MMADHC KO K562 cells treated with or without antimycin+oligomycin for three days.
